# Inflammation-Related Biomarkers for the Prediction of Prognosis in Colorectal Cancer Patients

**DOI:** 10.3390/ijms22158002

**Published:** 2021-07-27

**Authors:** Takehito Yamamoto, Kenji Kawada, Kazutaka Obama

**Affiliations:** 1 Department of Surgery, Graduate School of Medicine, Kyoto University, Kyoto 606-8501, Japan; takehito_y@kuhp.kyoto-u.ac.jp (T.Y.); kobama@kuhp.kyoto-u.ac.jp (K.O.); 2 Department of Gastroenterological Surgery, Kitano Hospital, The Tazuke Kofukai Medical Research Institute, Osaka 530-8480, Japan

**Keywords:** inflammation-related marker, colorectal cancer, prognostic biomarker

## Abstract

Colorectal cancer (CRC) is the leading cause of cancer deaths around the world. It is necessary to identify patients with poor prognosis or with high risk for recurrence so that we can selectively perform intensive treatments such as preoperative and/or postoperative chemotherapy and extended surgery. The clinical usefulness of inflammation-related prognostic biomarkers available from routine blood examination has been reported in many types of cancer, e.g., neutrophil–lymphocyte ratio (NLR), lymphocyte–C-reactive protein ratio (LCR), platelet–lymphocyte ratio (PLR), lymphocyte–monocyte ratio (LMR), and so on. Moreover, some scoring systems based on circulating blood cell counts and albumin concentration have been also reported to predict cancer patients’ prognosis, such as the Glasgow prognostic score (GPS), systemic inflammation score (SIS), and prognostic nutritional index (PNI). The optimal biomarker and optimal cutoff value of the markers can be different depending on the cancer type. In this review, we summarize the prognostic impact of each inflammation-related marker in CRC.

## 1. Introduction

Colorectal cancer (CRC) is one of the main causes of cancer death in the world. Despite recent improvements in multidisciplinary approaches, including surgery, chemotherapy, and radiotherapy, the mortality rate of CRC is still high, especially in patients with distant metastasis or postoperative recurrence even after curative surgery. Development of the optimal biomarker useful for the prediction of recurrence or poor prognosis is clinically important in order to identify patients who can benefit from intensive treatment including chemotherapy, chemoradiotherapy, and extended surgery.

**Figure 1:**
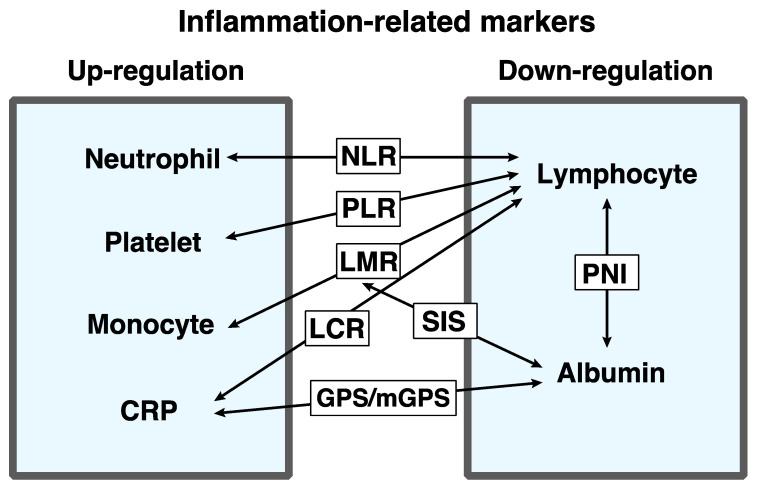
Overview of the inflammation-related markers. Inflammation-related serum parameters can be classified into the two groups: upregulation variables in disease progression (neutrophil, platelet, monocyte, and C-reactive protein (CRP)), and downregulation variables in disease progression (lymphocyte and albumin). The combination of the two of them can be used as inflammation-related markers.

The cancer-associated systemic inflammatory response is one of the critical indicators of tumor progression. Numerous previous studies have reported serum systemic inflammatory markers that can be useful for the prediction of prognosis, e.g., neutrophil–lymphocyte ratio (NLR), lymphocyte–monocyte ratio (LMR), platelet–lymphocyte ratio (PLR), Glasgow prognostic score (GPS), lymphocyte–C-reactive protein ratio (LCR), systemic inflammation score (SIS), and prognostic nutritional index (PNI) (Figure 1). These markers are easily available by use of routine blood examinations for cancer patients.

However, the best combination and optimal cutoff value can be different depending on the cancer type. Moreover, various anti-cancer treatments can differently affect the systemic inflammation status. Therefore, it is mandatory to focus on a certain cancer type so that we can evaluate the prognostic impact of inflammation-related markers.

Previously, we reported that the combination of GPS and NLR could effectively predict the prognosis of CRC patients who underwent curative resection [1]. In addition, we focused on rectal cancer patients (Stage II and III) and showed that the combination of circulating lymphocyte count and serum albumin concentration could be a useful predictor for prognosis [2]. However, there are many other inflammation-related biomarkers investigated regarding CRC. Thus, in the present report, we review previous papers about inflammation-related biomarkers focusing on CRC.

## 2. Neutrophil-Related Markers

The cancer-associated systemic inflammatory response is often correlated with an increase in circulating neutrophil counts. Neutrophils secrete cytokines and chemokines, which play important roles in cancer progression. Yang et al. reported that a high serum neutrophil count itself was independently associated with poor overall survival (OS) and progression-free survival (PFS) in metastatic CRC patients with wild-type RAS [3]. On the other hand, lymphocytes can promote a cytotoxic immune response to cancer. Some previous papers reported that a decrease in serum lymphocyte counts negatively affected the prognosis of CRC patients [3,4,5,6]. Noh et al. analyzed 231 colon cancer patients who underwent curative resection and adjuvant chemotherapy, and found that high post-chemotherapy lymphocyte counts (≥1.78 × 10^9^/L) were associated with better disease-free survival (DFS) [6].

The neutrophil–lymphocyte ratio (NLR) is one of the most robust biomarkers to predict prognosis in various types of cancer. Proctor et al. analyzed 12,118 patients including 1413 CRC patients, and indicated that NLR was a significant surrogate marker for OS and cancer-specific survival (CSS) [7]. There were numerous reports about NLR that focused on CRC (Table 1). The cutoff values were different depending on papers; the smallest one was 2, while the largest was 5, and they were determined in a different manner, i.e., by use of receiver-operating characteristic (ROC) curve, referring to previous reports, or using the median/mean value among the study population. Li et al. enrolled the most CRC patients (*n* = 5336) following curative resection (Stage I-III) and reported that NLR > 2.72 was an independent predictor for OS and DFS [8].

The patient population enrolled in each study was also different, i.e., CRC patients who underwent curative resection, metastatic CRC (Stage IV), colorectal liver metastasis (CRLM) following liver resection, CRC with peritoneal carcinomatosis, and so on. For example, Dell’Aquila et al. analyzed 413 metastatic CRC who received FOLFOXIRI or FOLFIRI plus bevacizumab enrolled in a TRIBE trial [55] and reported that NLR > 3.0 independently affected OS but not PFS [40]. Feliciano et al. analyzed 2470 Stage I-III CRC patients who underwent curative resection and found that the combination of high NLR (≥3.0) and sarcopenia determined by computed tomography scan was an independent predictor for OS [30].

Most of the papers evaluated “preoperative NLR”, while some assessed NLR in a different way. Yasui et al. stratified 568 CRC patients into three groups depending on inflammation status: preoperatively low (normal group), preoperatively high but postoperatively low (normalized group), and persistently high (elevated group) [52]. They indicated that the normal group showed a better prognosis than the elevated group regarding several inflammation-related markers, including NLR. Similarly, Li et al. also focused on the change between pre- and post-operative NLR [38]. They defined “post-NLR minus pre-NLR” as the delta-NLR and reported that the delta-NLR ≥ 0 group had better prognosis than the delta-NLR < 0 group, and that delta-NLR independently affected OS in CRC patients who underwent curative resection (Stage I-III). Chan et al. analyzed 2280 CRC patients who underwent surgery and found that the combination of pre- and post-NLR was a significant predictor for OS, i.e., the patients with high preoperative and high postoperative NLR (>3.75) exhibited the worst OS, whereas those with low preoperative and low postoperative NLR (≤3.75) exhibited the best OS [39].

On the other hand, Thiagarajan et al. analyzed 436 CRC patients with peritoneal carcinomatosis, and evaluated NLR on several postoperative timings (0, 1 to 3, 4 to 7, 8 to 21, 22 to 56, and 57 to 90 days) [53]. They reported that high NLR (postoperative days 8–21) >13.26 was most strongly associated with poor OS, while high NLR (postoperative 57 to 90) >1.57 was most strongly associated with poor RFS.

Some previous reports indicated that NLR could be predictive for recurrence patterns after liver resection against CRLM. Giakoustidis et al. reported that the patients with preoperatively elevated NLR (>2.5) had a higher risk for extrahepatic/multifocal recurrence [23]. Similarly, Verter et al. indicated that a high NLR (>3) was significantly associated with extrahepatic recurrence after surgery against CRLM [54].

Thus, numerous papers have proven that NLR can be used as a significant predictor for the survival of CRC patients, although the characteristics of the patients enrolled and the optimal cutoff values of NLR varied from report to report.

## 3. Albumin-Related Markers

Albumin is one of the acute-phase proteins and decreases in response to inflammation. Moreover, a low albumin concentration reflects cancer-induced malnutrition and can have negative impact on prognosis.

The GPS, introduced by Forrest et al., is a well-known inflammation-related marker that includes serum C-reactive protein (CRP) levels and serum albumin levels [56]. GPS is determined as follows: GPS of 2, CRP > 1.0 mg/dL and albumin < 3.5 g/dL; GPS of 1, either CRP > 1.0 mg/dL or albumin < 3.5 g/dL; and GPS of 0, neither CRP > 1.0 mg/dL nor albumin < 3.5 g/dL. Namely, high GPS reflects both systemic inflammation (elevated CRP) and low nutritional state (hypoalbuminemia).

A number of previous studies have revealed the postoperative prognostic potential of GPS (Table 2). As mentioned above, we previously reported that a high score of GPS was significantly associated with CSS and DFS from the analysis of 448 CRC patients who underwent curative resection [1]. Lee et al. also analyzed 1590 CRC patients who underwent curative resection, and found that a GPS of 1 or 2 independently affected survival [57].

GPS is also useful for metastatic CRC. Kobayashi et al. collected data from 63 CRLM patients who underwent curative resection and reported that GPS 1 or 2 was an independent prognostic factor for CSS [61]. Similarly, Kobayashi et al. investigated 99 CRC patients with lung metastasis following resection and indicated that GPS was an independent predictor for OS [62].

GPS has been modified by numerous recent reports so that it could be more sensitive for the prediction of prognosis; this is often called “modified GPS” (mGPS) (Table 3). For example, Park et al. constructed mGPS as follows: CRP ≤ 1.0 mg/dL is classified as a score of 0, CRP > 1.0 mg/dL and albumin ≥ 3.5 g/dL is a score of 1, and a CRP > 1.0 mg/dL and albumin < 3.5 g/dL is a score of 2. They analyzed 1000 CRC patients who underwent curative surgery and proved that the combination of mGPS and TNM stage was useful for the prediction of postoperative prognosis [63]. Guthrie et al. adopted the same definition of mGPS as Park et al. [63] and found that both preoperative and postoperative mGPS independently could affect the CSS of 206 CRC patients who underwent curative surgery [15].

On the other hand, Inoue et al. defined mGPS as follows: CRP ≤ 0.5 mg/dL and albumin ≥ 3.5 mg/dL was a score of 0, CRP > 0.5 mg/dL or albumin < 3.5 mg/dL was a score of 1, and CRP > 0.5 mg/dL and albumin < 3.5 mg/dL was a score of 2. They indicated the prognostic potential of this mGPS by analyzing 245 Stage IV and recurrent CRC patients treated with chemotherapy [67].

Thus, there were a number of different mGPSs, and the optimal definition is still controversial.

Other than GPS, there are also several albumin-related prognostic markers. For example, the prognostic nutritional index (PNI), first reported by Onodera et al. [71], is a well-known prognostic marker in Japan. PNI is calculated as “albumin level (g/L) + 0.005 × lymphocyte count”, and decreased PNI can affect cancer-related prognosis. Tokunaga et al. evaluated 468 CRC patients who underwent curative resection and indicated that PNI was independently associated with OS and RFS, and that PNI was more sensitive than mGPS if combined with TNM stages [69]. They adopted the cutoff value of 45 of PNI. Similarly, Mohri et al. analyzed 365 CRC patients who underwent curative resection, and reported that PNI < 45 independently affected OS [72]. On the other hand, Tominaga et al. established the optimal cutoff value of PNI as 42.4 by using ROC analysis [73]. They evaluated 84 elderly CRC patients (≥85 years) who underwent curative resection and reported that low PNI was independently associated with OS and RFS.

We have recently reported the novel prognostic marker “LA”, which means “lymphocyte count × albumin level (g/dL) [2]. We retrospectively collected data from a total of 448 rectal cancer patients (Stage II and III) from two large centers, and found that low LA (≤5950) was independently associated with both OS and RFS. Theoretically, LA is similar to PNI but LA is more easily calculated and simply used for the stratification of patients. However, our cohort was focused on rectal cancer patients and whether it can be applied to colorectal cancer patients as a whole remains to be elucidated.

Wang et al. analyzed 877 CRC patients who underwent curative resection (Stage I-III) and built a scoring system, “NLR–albumin”. They reported that the combination of high NLR (≥2.39) and hypoalbuminemia (<39.75 g/L) was a negative prognostic factor for OS in the multivariate analysis [74]. Hong et al. named “NLR–albumin” as IPI (inflammation related prognostic index) and evaluated the prognostic impact of IPI [75]. In their definition, patients with both NLR ≤ 3.0 and albumin ≥ 35 g/L were allocated an IPI score of 0, patients with either NLR ≤ 3.0 or albumin < 35 g/L were given a score of 1, and patients with both NLR > 3.0 and albumin < 35 g/L were given a score of 2. They evaluated 571 CRC patients following curative resection and indicated that IPI was a statistically better predictor for CSS than mGPS.

Furthermore, Shibutani et al. showed the usefulness of ALI (advanced lung cancer inflammation index) for CRC patients [76]. ALI was calculated as BMI × albumin concentration/NLR. They set 28.9 as the cutoff value for ALI by using ROC analysis and reported that low ALI independently affected OS, based on the retrospective analysis for 159 unresectable metastatic CRC patients. As described above, sarcopenia has a negative impact on cancer-related prognosis. Low BMI is related to the sarcopenic status. Therefore, ALI can be a more sensitive marker than IPI, although the calculation is somewhat complicated.

The albumin–globulin ratio (AGR) is also reported as contributive for the prediction of cancer-related prognosis. Low AGR reflects the combination of hypoalbuminemia and hyperglobulinemia. Hyperglobulinemia is also correlated to systemic inflammation, which can promote cancer development. Li et al. investigated 5336 CRC patients who underwent curative resection (Stage I-III) and indicated that AGR was an independent factor of OS and DFS [8]. Similarly, Fujikawa et al. conducted a retrospective analysis for 248 colon cancer patients who underwent curative resection (Stage I-III) and found that AGR was independently associated with OS and DFS [77].

## 4. Monocyte-Related Markers

In the tumor microenvironment, monocytes play an important role in tumor progression. Monocytes differentiate into tumor-associated macrophages (TAMs) and can contribute to tumor infiltration and metastasis. An increase in serum monocyte count can reflect the activity of TAMs.

Zhang et al. reported that an elevated monocyte count (>595/mm^3^) was significantly associated with poor OS and DFS in a cohort of 270 pathological T3N0M0 rectal cancer patients who underwent curative resection [78]. Sasaki et al. reported that an elevated monocyte count (>300/mm^3^) was independently associated with poor CSS in a cohort of 97 CRLM patients who underwent liver resection [79]. Haruki et al. also investigated 64 CRLM patients who underwent liver resection and indicated that an increase in serum monocyte count of less than two times before and after surgery was independently associated with DFS [80]. Thus, the monocyte count itself can be an important predictor for prognosis.

The prognostic potential of the lymphocyte–monocyte ratio (LMR) has been evaluated in several previous reports (Table 4); that is, lower LMR, which means lower lymphocyte counts and higher monocyte counts, can reflect an active inflammation status.

Chan et al. evaluated 1623 CRC patients who underwent curative surgery (all stages) and found that elevated LMR (>2.38) was independently associated with better OS, although NLR and PLR were not [83]. They also indicated that the rate of histologically high-grade tumors was higher in the patients with low LMR than in those with high LMR, and that the tumors with low LMR were more likely to be found in the left-sided colon. Li et al. investigated 5336 CRC patients with curative resection (Stage I-III) and reported that lower LMR (≤2.83) was independently associated with worse OS and DFS [8].

Dolan et al. compared the prognostic value between composite ratios and cumulative scores of inflammation markers [85]. They devised a “lymphocyte–monocyte score (LMS)” as follows: patients with lymphocyte count ≥1.5 × 10^9^/L and monocyte count ≤0.80 × 10^9^/L were allocated a score of 0, patients with lymphocyte count <1.5 × 10^9^/L and monocyte count >0.80 × 10^9^/L were assigned a score of 2, and the others were assigned a score of 1. They reported that both LMR and LMS were significantly associated with OS and CSS in multivariate analysis. On the other hand. Chan et al. evaluated the perioperative change in inflammation markers in CRC patients and found that the patients with consistently high LMR (>2.86) both before and after surgery had significantly better prognosis [39].

SIS is a comparatively new prognostic marker that was first introduced by Chang et al. in patients with renal cell carcinoma [86]. SIS consists of serum albumin level and LMR. SIS stratifies patients as follows: patients with albumin > 4.0 g/dL and LMR > 4.44 were classified as a score of 0, patients with albumin < 4.0 g/dL and LMR < 4.44 were given a score of 2, and the others were given a score of 1. Suzuki et al. evaluated 727 CRC patients who underwent curative resection (all stages), and reported that both increased SIS and increased mGPS scores were independently associated with poorer prognosis [70]. Furthermore, they compared the predictive performance for prognosis between SIS and mGPS by the use of the time-dependent ROC curve, and found that SIS was superior to mGPS for the prediction of OS.

## 5. CRP-Related Markers

CRP is one of the most useful parameters to evaluate the inflammation status of cancer patients preoperatively or postoperatively. In many countries, however, CRP is not routinely tested in general clinical settings, although not in Japan; CRP is evaluated in almost all cases in a routine blood examination in Japan. Table 5 shows 12 previous papers that investigated the prognostic potential of CRP-related markers for CRC patients, 11 of which were reported by Japanese researchers.

CRP itself has been reported as a prognostic marker for CRC patients. For example, Koike et al. evaluated 300 CRC patients and reported that preoperative high CRP (>0.5 mg/dL) was an independent predictor of prognosis [87].

The combination of higher CRP and hypoalbuminemia can be also a sensitive biomarker for cancer-related prognosis. Matsuoka et al. evaluated 133 Stage III CRC patients who underwent curative resection and found that postoperative C-reactive protein–albumin ratio (CAR) (≥0.035) was significantly associated with worse OS and RFS [90]. Ide et al. analyzed 115 rectal cancer patients following neoadjuvant chemoradiothrapy (nCRT) and surgery (Stage I-III), and indicated that CAR ≥ 0.049 before CRT was an independent prognostic factor for OS and DFS [88]. Dolan et al. analyzed 801 CRC patients following curative resection (Stage I-III) and reported that CAR > 0.22 was one of the independent predictors for OS and CSS [36].

Lymphocyte–C-reactive protein ratio (LCR) is the combination of lymphocyte count and CRP, and is also a robust prognostic marker. Suzuki et al. investigated 16 inflammation-related markers, namely NLR, LMR, PLR, CAR, PNI, LCR, neutrophil–albumin ratio (NAR), monocyte–albumin (MAR), platelet–albumin ratio (PAR), neutrophil × monocyte, neutrophil × platelet, neutrophil × CRP, monocyte × platelet, monocyte × CRP, platelet × CRP, and LA, for 1303 CRC patients who underwent curative resection (Stage II–III) [91]. They indicated that LCR (≤12,980) was most significantly and independently correlated with worse OS and DFS. Okugawa et al. also found the prognostic potential of LCR in the discovery cohort (*n* = 373) and validated the result in the validation cohort (*n* = 104) [89]. They proved that low LCR (≤6000) was independently related to worse OS and DFS. Of note, they found that low LCR was also a significant predictor for postoperative infectious complication, which means that LCR can be a useful biomarker for both short- and long-term postoperative outcomes of CRC patients.

Most of the studies about CRP-related makers adopted preoperative parameters. However, as mentioned above, Yasui et al. analyzed the inflammation status between before and after surgery, and showed that postoperative, but not preoperative, inflammation-based markers (i.e., CAR, LCR, and NLR) were significantly associated with OS and RFS [52]. The best timing for the evaluation of CRP-related markers remains to be elucidated.

## 6. Platelet-Related Markers

As with neutrophils, platelets are also a typical blood cell component responsible for the inflammatory response, and thrombocytosis is often observed in solid tumor patients with chronic inflammation [93,94]. In the tumor microenvironment, platelets can promote tumorigenesis by facilitating angiogenesis by releasing proangiogenic proteins such as the vascular epidermal growth factor and transforming growth factor-beta. Platelet-derived growth factor, produced by platelets, also play an important role in promoting tumor growth and invasion. Furthermore, cytokines and chemokines produced by platelets can promote cancer-associated inflammation.

In several previous reports, serum platelet count was reported to be a prognostic factor in CRC. Ishizuka et al. analyzed 453 CRC patients (all stages) who underwent surgery and reported that an elevated platelet count (>300 × 10^9^/L) was independently associated with poor OS [95]. Similarly, Pedrazzani et al. indicated that an elevated platelet count (>350 × 10^9^/L) could be a significant predictor for poor OS and CSS in Stage IV CRC patients [34].

Platelet–lymphocyte ratio (PLR) has been reported as a well-known prognostic marker in CRC (Table 6). A high PLR reflects both an increase in platelet count and a decrease in lymphocyte count.

Kim et al. investigated 1868 CRC patients and reported that high PLR (≥160) and high NLR (≥3.0) were independent predictors for poor OS and DFS in Stage III and IV cases, although not in Stage I and II cases [32]. Erstad et al. evaluated 151 patients with CRLM who underwent liver resection and found that PLR ≥ 220 and NLR ≥ 5 were independent factors that negatively affected OS [47].

Mercier et al. recently introduced a new prognostic marker, the platelet–neutrophil–lymphocyte ratio (PNLR) [96]. High PNLR reflects an increase in both platelet and neutrophil counts. They analyzed 305 metastatic CRC patients and indicated that high PNLR (≥2000) was significantly associated with poor OS and RFS in multivariate analysis. Chen et al. named PNLR as the systemic immune–inflammation index (SII) and evaluated the prognostic impact of this marker in 1383 CRC patients who underwent surgery [97]. They indicated that SII was an independent predictor for OS and that the diagnostic performance of SII was superior to that of NLR and PLR.

## 7. Conclusions

Accumulating evidence has shown the usefulness of inflammation-related biomarkers in CRC patients. In this review, we divided the markers into five categories: neutrophil-related markers, albumin-related markers, monocyte-related markers, CRP-related markers, and platelet-related markers. Of note, the characteristics of the enrolled patients and the cutoff values of each inflammation-related marker varied widely from study to study. Multicenter prospective studies will be required to find the optimal cutoff values.

## Figures and Tables

**Table 1:** Neutrophil–lymphocyte ratio (NLR) in previous reports on colorectal cancer.

Year	Author	Population	Patient (*n*)	Cutoff
2008	Halazun et al. [9]	CRLM following liver resection	440	5
2009	Kishi et al. [10]	CRLM following liver resection or chemo alone	290	5
2010	Ding et al. [11]	colon cancer following curatice resection (Stage II)	141	4
2011	Hung et al. [12]	colon cancer following curatice resection (Stage II)	1040	5
2011	Proctor et al. [7]	all cancer (1413 CRC)	12,118	4
2012	Chiang et al. [13]	CRC following curative resection (Stage I–III)	3857	3
2012	Carruthers et al. [14]	rectal cancer following nCRT(Stage II–IV)	115	5
2013	Guthrie et al. [15]	CRC following surgery (all stages)	206	5
2013	Mallappa et al. [16]	CRC following surgery (all stages)	297	5
2013	Krauthamer et al. [17]	rectal cancer following CRT (Stage II–III)	140	5
2014	Malietzis et al. [18]	CRC following curative resection (Stage I–III)	506	3
2014	Kim et al. [19]	rectal cancer following nCRT and surgery (Stage I–III)	102	3
2014	Shen et al. [20]	rectal cancer following nCRT and surgery (Stage I–III)	199	2.8
2015	Choi et al. [21]	CRC following curative resection (Stage I–III)	549	2.6
2015	Shibutani et al. [22]	CRC following curative resection (Stage II–III)	254	2.5, 3
2015	Giakoustidis et al. [23]	CRLM following curative resection	169	2.5
2015	Neal et al. [24]	CRLM following curative resection	302	5
2015	Tohme et al. [25]	unresectable metastatic CRC following radioembolization	104	5
2015	Nagasaki et al. [26]	rectal cancer following nCRT and surgery	201	3
2016	Li et al. [8]	CRC following curative resection (Stage I–III)	5336	2.72
2016	Ishizuka et al. [27]	CRC following surgery (all stages)	627	2.9
2016	Zou et al. [28]	CRC following surgery (all stages)	216	4.98
2017	Song et al. [29]	CRC following curative resection (all stages)	1744	2
2017	Feliciano et al. [30]	CRC following curative resection (Stage I–III)	2470	3
2017	Oh et al. [31]	CRC following curative resection (Stage II)	261	2.6
2017	Kim et al. [32]	CRC following surgery (all stages)	1868	3
2017	Balde et al. [33]	CRC following surgery (all stages)	280	3.5
2017	Pedrazzani et al. [34]	CRC following surgery (all stages)	603	3.5
2018	Dimitriou et al. [35]	CRC following curative resection (Stage I–III)	296	4.7
2018	Dolan et al. [36]	CRC following curative resection (Stage I–III)	801	3, 5
2018	Patel et al. [37]	CRC following curative resection (Stage I–III)	972	5
2018	Li et al. [38]	CRC following curative resection (Stage I–III)	616	3
2018	Chan et al. [39]	CRC following surgery (all stages)	2280	3.75
2018	Dell’Aqui et al. [40]	metastatic CRC following chemotherapy (TRIBE trial)	413	3
2018	Ward et al. [41]	rectal cancer following nCRT and surgery (Stage II–III)	146	4.47
2019	Inamoto et al. [1]	CRC following curative resection (all stages)	448	2.05
2019	Climent et al. [42]	CRC with dMMR following curative resection (Stage I–III)	566	5
2019	Dupre et al. [43]	CRLM following liver resection	343	many
2019	Mao et al. [44]	CRLM following NAC and curative resection	183	2.3
2019	Dudani et al. [45]	rectal cancer following nCRT and surgery (Stage II–III)	1237	4
2020	Mazaki et al. [46]	CRC following curative resection (Stage II–III)	375	3
2020	Erstad et al. [47]	CRLM following liver resection	151	5
2020	Cimino et al. [48]	CRLM following NAC and curative resection	128	2.12
2020	Yoshida et al. [49]	rectal cancer following curative resection (Stage I–II)	130	2.58
2020	Zhang et al. [50]	rectal cancer following nCRT and surgery (Stage II–III)	472	2.3
2020	Xia et al. [51]	T1-2 rectal cancer following curative resection	154	2.8
2021	Yasui et al. [52]	CRC following curative resection (Stage III)	563	2.39
2021	Thiagarajan et al. [53]	CRC following peritoneal carcinomatosis	436	many
2021	Verter et al. [54]	CRLM following liver resection	231	3

CRLM: colorectal liver metastasis, CRC: colorectal cancer, RFA: radiofrequency ablation, nCRT: neoadjuvant chemoradiotherapy, dMMR: DNA mismatch repair deficiencies, NAC: neoadjuvant chemotherapy.

**Table 2:** Glasgow prognostic scale (GPS) in previous reports on colorectal cancer.

Year	Author	Population	Patient (*n*)
2007	Ishizuka et al. [58]	CRC following surgery (all stages)	315
2013	Ishizuka et al. [59]	CRC following primary resection (Stage IV)	108
2014	Choi et al. [60]	CRC following surgery (all stages)	105
2015	Shibutani et al. [22]	CRC following curative resection (Stage II–III)	254
2016	Ishizuka et al. [27]	CRC following surgery (all stages)	627
2019	Inamoto et al. [1]	CRC following curative resection (all stages)	448
2020	Lee et al. [57]	CRC following curative resection (all stages)	1590

CRC: colorectal cancer, CRLM: colorectal liver metastasis.

**Table 3:** Modified Glasgow prognostic scale (mGPS) in previous reports.

Year	Author	Population	Patient (*n*)
2007	Leitch et al. [64]	CRC following curative resection (all stages)	149
2009	Roxburgh et al. [65]	CRC following curative resection (Stage I–III)	287
2011	Toiyama et al. [66]	CRC following curative resection (Stage II–III)	219
2013	Guthrie et al. [15]	CRC following surgery (all stages)	206
2013	Inoue et al. [67]	Stage IV/recurrent CRC treated by chemotherapy	245
2016	Park et al. [63]	CRC following curative resection (all stages)	1000
2017	Park et al. [68]	CRC following curative resection (Stage I–III)	331
2017	Tokunaga et al. [69]	CRC following curative resection (Stage I–III)	468
2018	Suzuki et al. [70]	CRC following curative resection (all stages)	727
2018	Dolan et al. [36]	CRC following curative resection (Stage I–III)	801

CRC: colorectal cancer.

**Table 4:** Lymphocyte–monocyte ratio (LMR) in previous reports on colorectal cancer.

Year	Author	Population	Patient (*n*)	Cutoff
2014	Stotz et al. [81]	CRC following curative resection (Stage II–III)	372	2.83
2015	Shibutani et al. [82]	unresectable metastatic CRC	104	3.38
2016	Li et al. [8]	CRC following curative resection (Stage I–III)	5336	2.83
2017	Chan et al. [83]	CRC following curative resection (Stage I–III)	1623	2.38
2018	Dolan et al. [36]	CRC following curative resection (Stage I–III)	801	2.4
2018	Chan et al. [39]	CRC following surgery (all stages)	2280	2.86
2018	Ward et al. [41]	rectal cancer following nCRT and surgery (Stage II–III)	146	2.86
2019	Chen et al. [84]	CRC with obstruction	128	1.67
2019	Dupre et al. [43]	CRLM following liver resection	343	many
2021	Yasui et al. [52]	CRC following curative resection (Stage III)	563	5.215
2021	Thiagarajan et al. [53]	CRC with peritoneal carcinomatosis	436	many

CRC: colorectal cancer, CRLM: colorectal liver metastasis, CRS: cytoreductive surgery, HIPEC: hyperthermic intraperitoneal chemotherapy.

**Table 5:** CRP-related markers in previous reports on colorectal cancer.

Year	Author	Markers	Population	Patient (*n*)	Cutoff
2008	Koike et al. [87]	CRP	CRC following surgery (all stages)	300	0.5
2016	Ishizuka et al. [27]	CAR	CRC following surgery (all stages)	627	0.038
2017	Ide et al. [88]	CAR	rectal cancer following CRT and surgery (Stage I–III)	115	0.049
2018	Dolan et al. [36]	CAR	CRC following curative resection (Stage I–III)	801	0.22
2019	Okugawa et al. [89]	LCR	CRC following surgery (all stages)	477	6000
2020	Matsuoka et al. [90]	CAR	CRC following curative resection (Stage III)	133	0.035
2020	Suzuki et al. [91]	LCR	CRC following curative resection (Stage II–III)	1303	12980
2021	Yasui et al. [52]	CAR	CRC following curative resection (Stage III)	563	0.025
2021	Yasui et al. [52]	LCR	CRC following curative resection (Stage III)	563	10424
2021	Taniai et al. [92]	CLR	CRLM following liver resection	197	62.8 × 10^−6^

CRP: C-reactive protein, CRC: colorectal cancer, CRLM: colorectal liver metastasis, nCRT: neoadjuvant chemoradiotherapy, CAR: C-reactive protein–albumin ratio, LCR: lymphocyte–C-reactive protein ratio, CLR: C-reactive protein–lymphocyte ratio.

**Table 6:** Platelet–lymphocyte ratio (PLR) in previous report on colorectal cancer.

Year	Author	Population	Patient (*n*)	Cutoff
2016	Zou et al. [28]	CRC following surgery (all stages)	216	246.36
2017	Kim et al. [32]	CRC (all stages)	1868	160
2017	Pedrazzani et al. [34]	CRC following surgery (all stages)	603	350
2018	Ward et al. [41]	rectal cancer following nCRT and surgery (Stage II–III)	146	203.6
2019	Dupre et al. [43]	CRLM following liver resection	343	many
2019	Dudani et al. [45]	rectal cancer following nCRT and surgery (Stage II–III)	1237	150
2020	Erstad et al. [47]	CRLM following liver resection	151	220
2021	Thiagarajan et al. [53]	CRC with peritoneal carcinomatosis	436	many

CRC: colorectal cancer, CRLM: colorectal liver metastasis, CRS: cytoreductive surgery, HIPEC: hyperthermic intraperitoneal chemotherapy, nCRT: neoadjuvant chemoradiotherapy.
